# Objective measurement of physical activity: improving the evidence base to address non-communicable diseases in Africa

**DOI:** 10.1136/bmjgh-2018-001044

**Published:** 2018-10-08

**Authors:** Anna Louise Barr, Elizabeth H Young, Manjinder S Sandhu

**Affiliations:** 1 Department of Medicine, University of Cambridge, Cambridge, UK; 2 Wellcome Sanger Institute, Genome Campus, Hinxton, UK

**Keywords:** physical activity, physical inactivity, objective physical activity, non-communicable diseases, Africa, physical activity interventions, determinants of physical activity

**Summary box**

- Physical inactivity is one of the major modifiable risk factors for non-communicable diseases (NCDs).
- African countries, many of which are in the early stages of an emerging NCD epidemic, have the opportunity to reduce the financial and health burden of NCDs through the early implementation of prevention strategies that promote physical activity.
- The design and success of physical activity promotion strategies requires an improved understanding of the physical activity patterns of African populations and their multiple determinants.
- Integrating objective physical activity measurement devices within research and routine surveillance infrastructures in Africa would improve the quality, coverage and resolution of physical activity data on the continent and inform the design and implementation of effective policies and interventions for the local context.
- This framework also permits the monitoring of population physical activity patterns and trends, as well as the evaluation of interventions at the individual and population level.

## Introduction

The benefits of physical activity are wide ranging and associated with reduced disease risk and improved mental health. Strong evidence from high-income countries (HICs) has revealed a clear dose–response relationship between physical activity and improved health status. Regular physical activity is associated with a reduced risk of coronary heart disease, high blood pressure, stroke, metabolic syndrome, type 2 diabetes, breast and colon cancer, depression and dementia.^1 2^ Similarly, in low-income and middle-income countries (LMICs), higher physical activity levels are associated with a lower risk of cardiovascular disease and mortality.^3^ Physical activity has also been associated with improved mental health outcomes and increased self-esteem, self-efficacy and social capital; however, the direction and strength of these relationships needs further investigation to fully understand the impact of physical activity on mental well-being.^4^

## Global burden of physical inactivity

Guidelines on the total volume of physical activity required to reduce the risk of specific NCDs are imprecise; however, the WHO recommends that adults undertake at least 150 min of moderate-intensity physical activity per week, or 75 min of vigorous-intensity physical activity per week, to reduce the overall risk of NCDs.^1^ Worldwide 23.3% of adults do not meet these recommendations and are considered physically inactive.^2^ Low physical activity was identified as one of the leading risk factors responsible for the global disease burden, with an estimated 1.4 million deaths attributed to low physical activity in 2016, an increase of 18.4% since 2006.^5^ The disease burden is especially high in LMICs, with 75% of the estimated 13.4 million disability-adjusted life years lost to physical inactivity in 2013 attributed to these regions.^6^ In the same year, physical inactivity was estimated to have cost global healthcare systems international $ 53.8 billion.^6^

## Burden of physical inactivity in Africa

Africa is a region in transition. The demographic profile of the continent is changing: reductions in infant and child mortality have contributed to rapid population growth and a concomitant rise in life expectancy. Africa is also the fastest urbanising continent, with the number of people living in urban areas projected to triple in the next 50 years.^7^ Globalisation, technological advances and economic development have all contributed to a trend towards sedentary lifestyles and the uptake of unhealthy behaviours on the continent. These demographic, social and environmental changes have led to an emerging NCD epidemic, complicated by an existing high prevalence and incidence of chronic infectious diseases such as tuberculosis, malaria and HIV. Physical activity is influenced by a complex interaction of biological, social and environmental factors. As such, the distribution and determinants of physical inactivity, and its relative contribution to the burden of NCDs, will likely differ within and between African countries at varying stages of economic development and with diverse sociocultural contexts. A meta-analysis of self-reported physical activity data from 22 African countries found 16% of men and 24% of women were physically inactive, which is comparable to prevalences reported in HICs.^8^ However, the prevalence of physical inactivity varied between countries, ranging from 46.2% of men and 60.3% of women in Mali to 3.8% of men and 4.2% of women in Mozambique.^8^ The source of this variation is not well understood, but differences in body mass index, perceptions of body image, socioeconomic status, urbanicity, safety, security and access to spaces that support physical activity are likely to be contributing factors.^9–15^

## Methods to measure physical activity

Current epidemiological evidence on the physical activity patterns of African populations and their relationship to NCDs is largely based on self-report questionnaires and a small number of studies using objective measurement devices. While self-report questionnaires are the most popular tool used to measure physical activity, particularly at scale, their validity often tends to be moderate-to-poor due to their reliance on participants accurately reporting their physical activity patterns.^16^ Discrepancies have previously been found between objectively measured and self-reported data. In one study conducted in the Seychelles, Ghana and South Africa, all men reported accumulating over 60 min of moderate-to-vigorous physical activity per day; however, when compared with accelerometery data only 32%, 56% and 58% of men accumulated 30 min or more of physical activity per day in each respective country.^17^ The differences for women were even greater.^17^ Furthermore, lower education may be associated with reduced reliability and validity estimates for certain physical activity measures in LMICs.^18 19^ Objective physical activity monitors, meanwhile, permit continuous data collection in real-life conditions at greater resolution for longer periods of time, improving the volume and accuracy of data available. Objective measurement devices, designed for academic purposes, have been used in large-scale population studies in HICs for over a decade, while commercial physical activity trackers and smart phone applications are increasingly being used in clinical trials and National Institutes of Health funded research.^20^ An optional protocol for the objective measurement of physical activity is being incorporated in the WHO STEPwise Approach to Chronic Disease Risk Factor Surveillance data collection tool, signalling the broad acceptance that these devices are an important resource in the epidemiologists’ toolkit.

## Improving our evidence base through the objective measurement of physical activity

The limitations of self-report data currently restrict our ability to accurately characterise the dose–response relationship between physical activity and disease risk in these populations. Meanwhile, the technology of objective measurement devices is constantly evolving: manufacturers are continually refining device algorithms to improve activity pattern recognition, increasing our ability to delineate the health benefits of specific activities.^9^ Our knowledge of the social, biological and environmental determinants of physical activity in Africa, including the impact of urbanisation and economic development, is limited by the need for context-specific measures for these determinants and the paucity of longitudinal data to determine causation.^21^ Smart phone ownership in Africa is accelerating: a median of 33% reported owning a smart phone in 2018, with a 14%–15% increase in ownership reported in Senegal, South Africa and Ghana between 2015 and 2017.^22^ This and the increasing availability of commercial physical activity monitors presents an opportunity to harness physical activity data collected as users carry out their daily lives.^22^ With careful consideration of ethical, data privacy and security issues, these devices have the potential to facilitate routine surveillance and monitoring of population physical activity patterns and trends, as well as evaluating clinical, societal and environmental interventions at the individual and population level.

## Opportunity for intervention

The majority of African countries have a policy or strategy which addresses physical inactivity, but only 70% report that these are operational,^23^ and evidence of effectiveness is scarce, partly due to the limited surveillance of physical activity across the continent. Integrating active living and sports programmes within schools and work places is a strategy advocated by global policy makers and a recommended ‘best buy’ intervention for the prevention and control of the global NCD pandemic.^24^ Likewise, creating environments which support the integration of physical activity into people’s daily lives through active travel or leisure activity is highly recommended.^25^ However, evidence of effective and sustainable interventions to increase physical activity at the population level within these settings is limited. Furthermore, the observed benefits of such interventions may not transfer directly to the African context. The unprecedented, and often uncontrolled, growth in urbanisation on the continent calls for urgent and innovative urban planning policies that support an active population.

Using context-specific data on population activity levels and their social and environmental determinants in the design and implementation of policies ensures they address the specific needs of populations in these regions. Similarly, integrating objective measurement of physical activity patterns and trends within research and surveillance infrastructure will be integral to improving the evidence base for policy development and the effective evaluation of these interventions at the population level. The success of such interventions will likely require engagement with education, commercial, urban planning, transportation, environmental and sports and recreation sectors. Consideration of individual-level factors such as wearer compliance and participation, as well as broader environmental issues such as climate, geography, safety and security will be necessary for the long-term sustainability of these interventions.^9 12 20 21 25^ Capacity building initiatives and financial support will also be integral to the widespread deployment of objective measurement devices. Finally, physical activity interventions should be integrated within a broader NCD prevention strategy addressing NCD risk factors such as diet, smoking and alcohol consumption.

## Conclusion

African countries are in a position to reduce the financial and health burden of the burgeoning NCD epidemic through the early implementation of prevention strategies targeting modifiable risk factors such as physical inactivity. Encouraging populations to regularly undertake physical activity requires a cultural shift: the benefits of physical activity to health and well-being need to be valued by both individuals and civil society. Governments and industrial partners have a pivotal role to play in realising these societal changes by adopting social and environmental policies and interventions that promote physical activity. Investing in objective measurement tools and physical activity surveillance infrastructure would improve the quality and coverage of physical activity data and ensure that clinical and public health decision making is guided by the best available evidence.
